# *ALK*^G1269A^ mutation as a potential mechanism of acquired resistance to crizotinib in an *ALK*-rearranged inflammatory myofibroblastic tumor

**DOI:** 10.1038/s41698-017-0004-3

**Published:** 2017-03-20

**Authors:** Sebastian Y. F. Michels, Andreas H. Scheel, Thomas Wündisch, Johannes M. Heuckmann, Roopika Menon, Michael Puesken, Carsten Kobe, Helen Pasternack, Carina Heydt, Matthias Scheffler, Rieke Fischer, Anne M. Schultheis, Sabine Merkelbach-Bruse, Lukas Heukamp, Reinhard Büttner, Jürgen Wolf

**Affiliations:** 1 Lung Cancer Group Cologne, University Hospital of Cologne, Cologne, Germany; 2 Department I of Internal Medicine, Center for Integrated Oncology, University Hospital of Cologne, Cologne, Germany; 3 Institute of Pathology, Center for Integrated Oncology, University Hospital of Cologne, Cologne, Germany; 4 Comprehensive Cancer Center Düsseldorf, University Hospital of Düsseldorf, Düsseldorf, Germany; 5 NEO New Oncology AG, Cologne, Germany; 6 Department of Radiology, Center for Integrated Oncology, University Hospital of Cologne, Cologne, Germany; 7 Department of Nuclear Medicine, Center for Integrated Oncology, University Hospital of Cologne, Cologne, Germany; 8 Institute of Hematopathology, Hamburg, Germany

## Abstract

Inflammatory myofibroblastic tumors are rare mesenchymal neoplasms frequently harboring oncogenic chromosomal rearrangements, most commonly, involving the *ALK* (anaplastic lymphoma kinase) gene. Treatment of this molecularly defined subgroup with the anaplastic lymphoma kinase inhibitor crizotinib has shown to be effective. However, comparable to lung adenocarcinoma, resistance inevitably develops. Second generation anaplastic lymphoma kinase inhibitors such as ceritinib are able to overcome acquired resistance to crizotinib. Here, we report the case of a patient with an inflammatory myofibroblastic tumors harboring a *DCTN1-ALK* fusion who developed resistance to crizotinib treatment. Next-generation sequencing of a rebiopsy sample revealed the acquisition of the *ALK* ^*G1269A*^ mutation as a mechanism of resistance. Therapy with ceritinib resulted in a short but profound clinical, metabolic and morphologic response. This case illustrates that (i) different tumor entities may share similar oncogenic driver mechanisms, rendering them vulnerable for the same therapeutic substances and (ii) likewise, the same mode of resistance may occur under targeted therapy among different tumor entities.

## Introduction

Inflammatory myofibroblastic tumors (IMTs) are rare mesenchymal neoplasms.^1^ IMTs predominantly affect young adults and prognosis is favorable upon surgical resection. Metastastic or recurrent disease is rare but is associated with poor prognosis most notably due to the lack of an effective systemic standard treatment.

IMTs are molecularly characterized by chromosomal rearrangements, most notably fusions of *ALK* (50% of cases), *ROS1* (ROS proto-oncogene 1) or *PDGFRβ* (platelet derived growth factor receptor beta).^2^ Efficacy of the kinase inhibitor crizotinib is proven in ALK-positive non-small cell lung cancer (NSCLC) and IMTs.^3, 4^ Second-generation ALK inhibitors are effective in crizotinib-resistant *ALK*-positive NSCLC, including cases with acquired *ALK* ^*G1269A*^ mutations.^5–7^

## Report

A 36-year-old female patient was first diagnosed in December 2009 with an *ALK*-rearranged pulmonary IMT (UICC stage: pT2 pN0 cM0 G2). Left pneumonectomy was performed (R0) but recurrence occurred in July 2011. Crizotinib treatment was initialized in October 2011 and resulted in a good radiologic response lasting until March 2014.

**Fig. 1:**
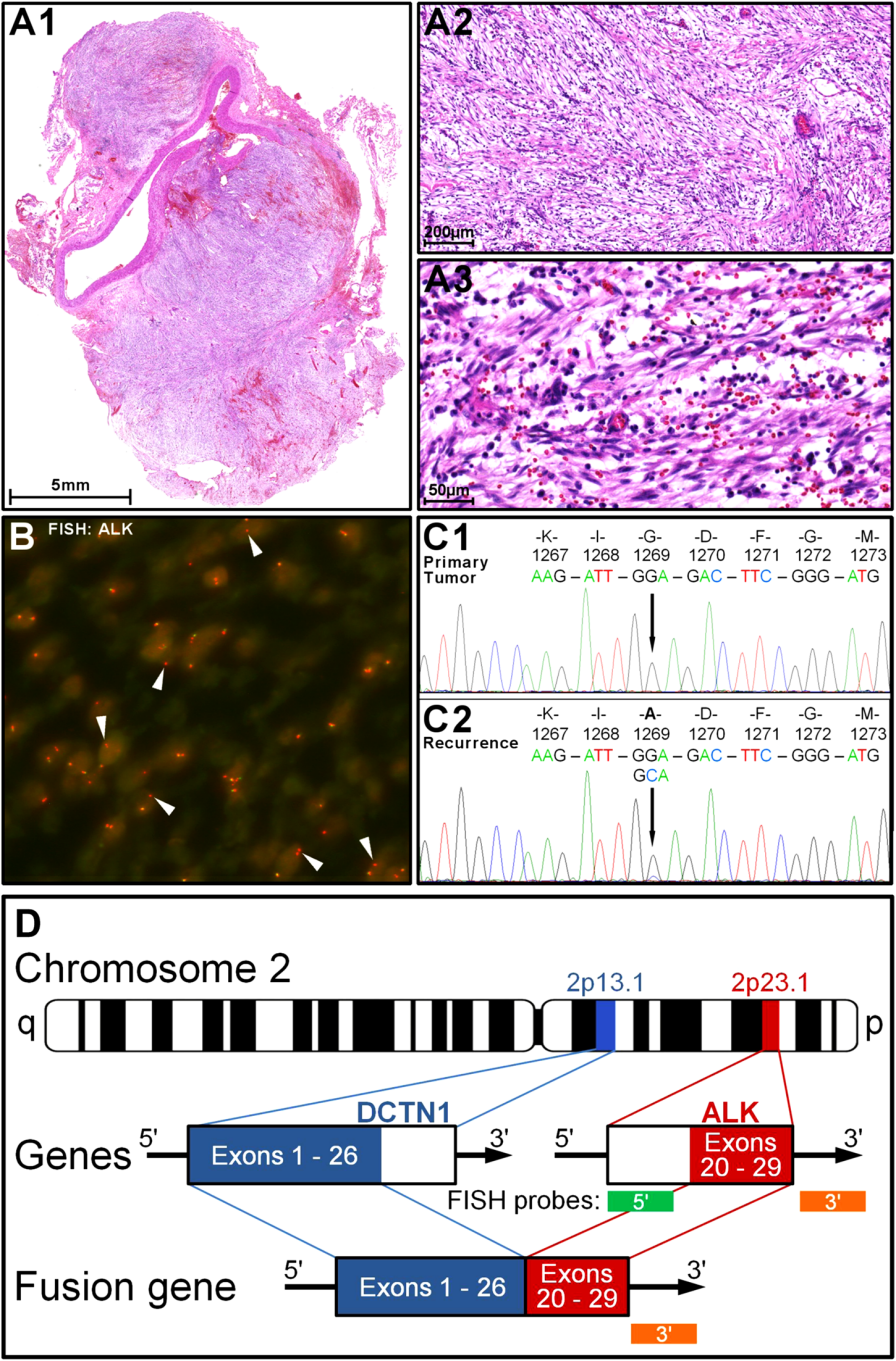
**a1**–**a3** Hematoxylin and eosin stained section of IMT. **b** Fluorescence in situ hybridization of post-crizotinib sample tissue, confirming rearrangement of *ALK* in 2p23: Isolated *red* signals (*arrows*) indicate loss of the 5' region of *ALK*. *Orange* fusion signals of the *red* 3' probe and the *green* 5' probe indicate normal *ALK* alleles. **c** Sanger sequencing of *ALK* of the initial tumor sample (C1) and the post-crizotinib sample (C2) identifying the *ALK* ^*G1269A*^ resistance mutation. **d** Schematic illustration of the *DCTN1-ALK* fusion. Exons 20–29 of the *ALK* gene, which contain the tyrosine kinase domain, are fused with exons 1–26 of the *DCTN1* gene. Both genes are located on chromosome 2p. The 5' region of *ALK* was apparently lost as indicated by the absence of the *green* FISH signal

**Fig. 2:**
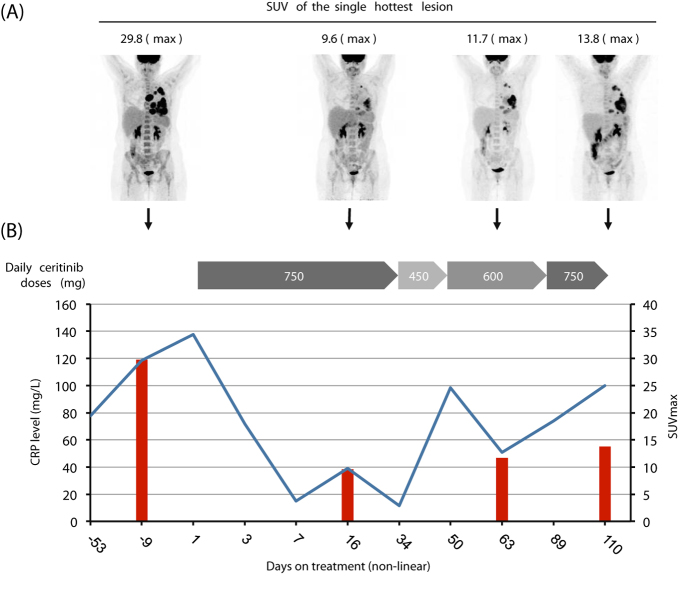
Metabolic response evaluation and CRP levels. **a** Baseline ^18^FDG-PET/CT and follow-up scans showing metabolic response with decreasing uptake. **b** Non-linear chart of serum CRP levels (mg/L) (*blue graph*) and change of SUV_max_ in the single hottest lesion (*red columns*). *Gray arrows*: ceritinib treatment at daily doses indicated (mg); *blue graph*: CRP levels (mg/L); *red columns*: SUV_max_ of the hottest lesion

At time of progression the patient presented with chills, night sweats, thoracic pain and significant elevation of serum C-reactive protein (CRP). Infection was ruled out. A rebiopsy confirmed the *ALK* fusion. Massively parallel (MPS) and hybrid-capture sequencing identified dynactin subunit 1 gene (*DCTN1*) as fusion partner and revealed the acquisition of an *ALK* ^*G1269A*^ mutation (confirmation by Sanger sequencing; Fig. 1). No other mutations were detected. Baseline ^18^FDG-PET/CT scan showed local progression and the appearance of new thoracic lesions (sum of target lesions: 122 mm; SUV_max_: 29.8; Fig. 2). Treatment with ceritinib was initiated in June 2014 (750 mg daily). Tumor-related symptoms resolved rapidly and serum CRP levels diminished. PET/CT at treatment day 17 revealed a metabolic response with a decrease of SUV_max_ by 68% and a reduction of the sum of target lesions by 25% (91 mm; SD). PET/CT at day 63 showed stable SUV_max_ and a reduction of target lesions by 30% (86 mm; unconfirmed partial response; Fig. 2). Subsequently CRP levels rose and tumor related symptoms recurred. Restaging revealed a progression according to RECIST with new lesions and an increased FDG-uptake (day 110).

The patient was discontinued from ceritinib at day 111 and deceased in February 2015.

## Discussion

To the best of our knowledge, this is the first report describing a molecular mechanism of acquired resistance to crizotinib in *ALK-*rearranged IMTs. DNA sequencing revealed the acquisition of *ALK* ^*G1269A*^ as the potential driver of resistance. All other investigated genes showed wild-type DNA sequences, highlighting the pivotal role of the *ALK* rearrangement in this case.

In our patient, treatment with ceritinib proved to be effective, however, for a short time only. Nevertheless, our case represents proof-of-concept for efficacy of second-generation ALK inhibitors in *ALK*-rearranged IMTs with acquired resistance to crizotinib. Several mutations, including *ALK* ^*G1269A*^, confer resistance to crizotinib in *ALK*-positive NSCLC and the next-generation ALK inhibitors ceritinib, alectinib, lolartinib, and brigatinib are able to overcome *ALK* ^*G1269A*^-driven resistance.^5–7^ At time of treatment of our patient, only ceritinib was available within an individual IND. Rebiopsy upon progression to ceritinib was not feasible and at that time third-generation inhibitors were not available.

In pre-clinical assays different *ALK* fusion partners and *EML4-ALK* fusion variants have shown to influence sensitivity to crizotinib.^8^ Whether, *DCTN1* which we identified as a novel translocation partner of *ALK* in IMTs has a negative impact on efficacy or duration of ALK inhibition is unclear.

CRP levels correlated inversely with tumor response and the elevation of inflammatory serum markers and their normalization following resection has regularly been observed in IMTs.^9^ CRP may therefor be a potential serum marker to follow treatment response.

This case illustrates how similar modes of resistance to ALK inhibitor treatment may occur in epithelial and mesenchymal malignancies, rendering them vulnerable to the same drugs. Although identical molecular targets do not generally confer equal sensitivity to the appropriate inhibitors in different tumors, our case raises the hope that the growing repertoire of targeted therapeutics will be effective against classes of malignancies defined by molecular alterations.

## Patient and methods

The patient was treated with ceritinib within an individual patient treatment program after the collection of the written informed consent.

Metabolic response was assessed using ^18^FDG-PET/CT according to the PET response criteria in solid tumors (PERCIST) v1.0 guideline, comparing SUV_max_ of the hottest single lesion in each consecutive scan (metabolic response definition: reduction in SUV_max_ of ≥30%). Morphologic response was assessed according to RECIST v1.1.

Fluorescence in situ hybridization was performed using *ALK*-specific dual-color break-apart probes (TriCheck®, Zytovision, Bremerhaven, Germany). MPS for *ALK* exons 21–25 and 13 more genes was performed on a MiSeq platform (Illumina, San Diego, USA).^10^ The hybrid-capture sequencing panel 'NEOplus' was used to test for point mutations, small insertions/deletions, copy number alterations, and fusions in 72 genes and to characterize the *ALK* rearrangement (NEO New Oncology AG, Cologne, Germany).
